# A study of the electro-haemodynamic coupling using simultaneously acquired intracranial EEG and fMRI data in humans

**DOI:** 10.1016/j.neuroimage.2016.08.001

**Published:** 2016-11-15

**Authors:** T. Murta, L. Hu, T.M. Tierney, U.J. Chaudhary, M.C. Walker, D.W. Carmichael, P. Figueiredo, L. Lemieux

**Affiliations:** a Dept. of Clinical and Experimental Epilepsy, UCL Institute of Neurology, London, United Kingdom; b Institute for Systems and Robotics and Department of Bioengineering, Instituto Superior Técnico, Universidade de Lisboa, Lisboa, Portugal; c Key Laboratory of Mental Health, Institute of Psychology, Chinese Academy of Sciences, Beijing, China; d UCL Institute of Child Heath, London, United Kingdom

**Keywords:** Intracranial EEG, BOLD, fMRI, Electro-haemodynamic coupling, Sharp wave

## Abstract

In current fMRI studies designed to map BOLD changes related to interictal epileptiform discharges (IED), which are recorded on simultaneous EEG, the information contained in the morphology and field extent of the EEG events is exclusively used for their classification. Usually, a BOLD predictor based on IED onset times alone is constructed, effectively treating all events as identical. We used intracranial EEG (icEEG)-fMRI data simultaneously recorded in humans to investigate the effect of including any of the features: amplitude, width (duration), slope of the rising phase, energy (area under the curve), or spatial field extent (number of contacts over which the sharp wave was observed) of the fast wave of the IED (the sharp wave), into the BOLD model, to better understand the neurophysiological origin of sharp wave-related BOLD changes, in the immediate vicinity of the recording contacts. Among the features considered, the width was the only one found to explain a significant amount of additional variance, suggesting that the amplitude of the BOLD signal depends more on the duration of the underlying field potential (reflected in the sharp wave width) than on the degree of neuronal activity synchrony (reflected in the sharp wave amplitude), and, consequently, that including inter-event variations of the sharp wave width in the BOLD signal model may increase the sensitivity of forthcoming EEG-fMRI studies of epileptic activity.

## Highlights

• First study of EEG morphology using simultaneous intracranial EEG-fMRI in humans
• The duration of sharp waves is significantly correlated with the BOLD signal amplitude.
• BOLD amplitude reflects more field potential duration than neuronal synchrony.
• Sharp wave duration should be included in BOLD models of epileptic discharges.

## Introduction

Blood oxygen level dependent (BOLD) functional magnetic resonance imaging (fMRI) is a non-invasive imaging technique commonly used to localise the neuronal activity underlying sensory, or cognitive functions, sleep, or rest (which may include epileptic activity), commonly captured by electrophysiological techniques (Murta et al., 2015). The fMRI whole-brain mapping capability complements the electroencephalogram (EEG) temporal richness, particularly relevant in epilepsy, providing localising information with potential clinical relevance. Simultaneously recorded scalp EEG and BOLD fMRI data have been used to locate brain regions involved in the generation and propagation of epileptic seizures (Chaudhary et al., 2012, Murta et al., 2012) and interictal epileptiform discharges (IED) (Caballero-Gaudes et al., 2013). The accurate delineation of these regions is the main purpose of the pre-surgical evaluation performed in patients with drug-resistant epilepsies because the best treatment available is to surgically disrupt them. IED are high-amplitude, fast electrophysiological transients constituted by a sharp wave (lasting up to 120 ms), often followed by a slow wave (lasting several hundreds of milliseconds) (De Curtis and Avanzini, 2001). Although commonly associated with the epileptic state, sharp waves have also been observed in the healthy hippocampus (Buzsáki et al., 1983, Suzuki and Smith, 1987, Skaggs et al., 2007). Such universality makes the sharp waves a potentially informative device for the study of the electrophysiological correlates of the BOLD signal.

Many studies investigated whether it is the continuous power of local field potentials (LFP), or the neuronal firing rate, that best predicts the BOLD signal (Logothetis et al., 2001, Mukamel et al., 2005, Nir et al., 2007). However, few have considered the morphology of the sharp wave as potentially informative to predict the amplitude BOLD of the signal; two studies in humans found mixed results regarding a significant correlation between the amplitudes of scalp EEG sharp waves and BOLD changes (Benar et al., 2002, LeVan et al., 2010); two studies in rats found a significant, positive correlation between the amplitude (and width (Geneslaw et al., 2011)) of LFP sharp waves and the amplitude of CBF changes (Geneslaw et al., 2011, Vanzetta et al., 2010). This limited number of studies is a lacuna in the field because the morphology of the sharp wave is likely to reflect interesting aspects of the underlying neuronal activity; its amplitude and rising phase slope are thought to reflect the degree of neuronal activity synchrony (Einevoll et al., 2013); its width (duration) and energy (area under the curve) are thought to reflect the duration of the underlying field potential (Gold et al., 2006); its spatial field extent (number of contacts over which the sharp wave was observed) is thought to reflect the extent of the surrounding, synchronised sources of neuronal activity (Einevoll et al., 2013).

Simultaneous intracranial EEG (icEEG)-fMRI data, recorded during the pre-surgical evaluation of patients with drug-resistant epilepsy, is now available (Carmichael et al., 2012, Boucousis et al., 2012), offering a unique opportunity to further investigate the relationship between these signals, especially at a local level (i.e. in the immediate vicinity of the activity generators). In terms of morphology, spatiotemporal localisation, and field distribution, sharp wave features are more accurately estimated using icEEG than scalp EEG. However, the comparatively greater amount and complexity of information related to sharp waves recorded on icEEG, poses a challenge to the modelling of the simultaneous BOLD changes. For example, in our data, sharp waves are observed at rates ranging from 0.17 to 1.7 events per second (see Table 1). For very regular and frequent events, the conventional way of modelling all events as identical can result in an essentially flat BOLD predictor; a very inefficient fMRI “experimental design” that potentially limits the sensitivity of the study (Dale, 1999, Friston et al., 1999). Modelling strategies that introduce some variability based on event-by-event feature measurements may actually increase the efficiency of the model, and also exploit the variability of both signals better.

We compared five sharp wave features (amplitude, slope of the rising phase, width, energy, and spatial field extent) in terms of their individual capability to explain variance of the BOLD signal amplitude that was not explained by the sharp wave onsets. Sharp wave features were quantified event-by-event, convolved with a haemodynamic kernel, and compared with the BOLD changes in the surroundings of the most active icEEG contact, within the General Linear Model (GLM) framework. Crucially, this choice of region of interest was used to ensure that we were studying the BOLD changes related to the generators rather than any potential propagation effects, which are commonly seen in epileptic activity. This study aimed to shed further light on the neurophysiological origin of the BOLD signal related to sharp waves, and improve the sensitivity of EEG-fMRI studies. To the best of our knowledge, this is the first study using invasively recorded sharp waves and simultaneous fMRI data to investigate which aspects of the sharp wave best explain the variance of the BOLD signal.

## Methods

We analysed icEEG and fMRI data simultaneously recorded from 6 patients with severe drug-resistant epilepsy, undergoing invasive EEG monitoring as part of their pre-surgical evaluation. This study was approved by the Joint UCL/UCLH Committees on the Ethics of Human Research, and the patients gave written informed consent.

### Simultaneous icEEG-fMRI data acquisition

MRI data was acquired on a 1.5 T scanner (TIM Avanto, Siemens, Erlangen, Germany), with a quadrature head transmit–receive RF coil using low specific absorption rate sequences (< 0.1 W/kg head average), simultaneously with icEEG data, in accordance with our acquisition protocol (Carmichael et al., 2012).

The patients were asked to lie still with eyes closed during data acquisition. One (in 3/6 cases) or two (3/6 cases) 10-min sessions of 200 fMRI volumes were acquired during rest.

The fMRI scan consisted of a gradient-echo EPI sequence with the following parameters: TR/TE/flip angle = 3000 ms/40 ms/90°, 64 × 64 acquisition matrix, 38 × 2.5 mm slices with 0.5 mm gap. In addition, a FLASH T1 weighted structural scan was acquired with the following parameters: TR/TE/flip angle = 15 ms/4.49 ms/25°, resolution 1.0 × 1.2 × 1.2 mm, FoV 260 × 211 × 170 mm, 256 × 176 × 142 image acquisition matrix with the readout direction lying in the sagittal plane; scan duration: 6 min 15 s.

The icEEG data were acquired with an MR-compatible system (Brain Products, Gilching, Germany) and related software (Brain Recorder, Brain Products, Gilching, Germany) at a 5 kHz sampling rate. The icEEG recording system was synchronised with the 20 kHz gradient MR scanner clock.

Computed tomography (CT) data were acquired shortly after the implantation of the icEEG electrodes and prior to the icEEG-fMRI acquisition.

### IcEEG and fMRI data pre-processing

SPM12 (http://www.fil.ion.ucl.ac.uk/spm) was used to realign and spatially smooth (using an isotropic 5 mm FWHM Gaussian kernel) the fMRI data. Prior to smoothing, physiological noise was removed from the fMRI data using FIACH (Functional Image Artefact Correction Heuristic) (Tierney et al., 2015).

MR acquisition-related artefacts were removed from the icEEG using an average template subtraction approach (Allen et al., 2000), and subsequently down-sampled to 500 Hz. Cardiac pulse-related artefact correction was not performed because the amplitude of this artefact in our icEEG recordings is much smaller (approximately 95%) than the amplitude of a typical IED (Carmichael et al., 2012).

IED quantification was performed on bipolar montage data; for depth electrodes, the signal from the contact of interest was subtracted from the adjacent medial one; for grid electrodes, the signal was subtracted from the adjacent anterior one.

### IED classification

**Fig. 1:**
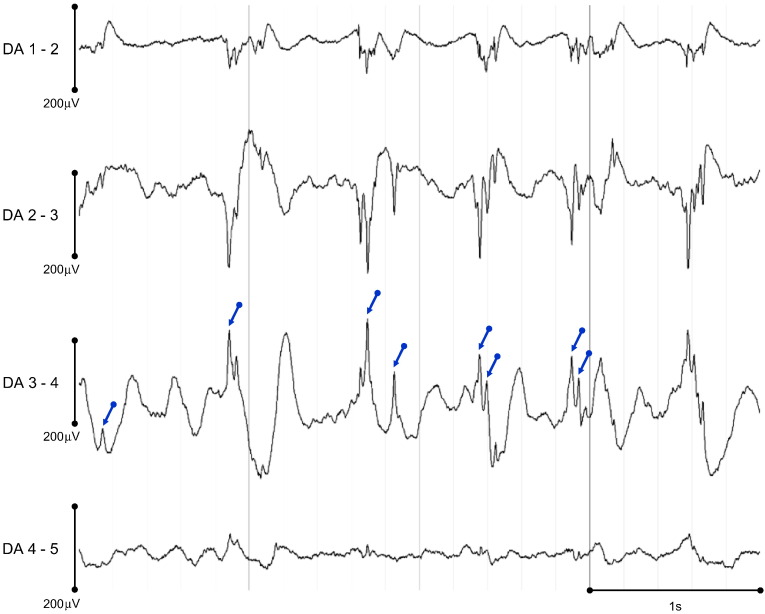
Representative EEG time courses (patient ID: E, events of interest set: *S*_*9*_, see Table 1) showing sharp waves of different amplitudes and widths (blue arrows point at events visually marked at COI: DA 3–4).

IED events (Fig. 1) were identified visually by an experienced EEG reviewer, and their onset time (peak of the sharp wave) was marked in relation to the start of the icEEG recording. IED were then grouped into classes, according to their spatiotemporal localisation and field distribution. Each IED class was labelled as either: *focal*, if simultaneously observed in 2–4 contiguous contacts; *regional*, if simultaneously observed in 5–10 contiguous contacts that could span up to two gyri; *widespread*, if involving > 10 contiguous contacts; or *non-contiguous*, if having a focal or regional field but also propagating to non-contiguous contacts. Additionally, the position of each manually placed IED marker was adjusted to the position of the peak value using an automated process applied on the 24 ms-wide window centred at the manual mark, because the accurate IED parameterisation is reliant on the precise marking of the IED peak. Visual inspection of the realignment results confirmed the validity of this approach.

### Patient-specific contacts of interest (COI)

For each focal IED class and contact pair, IED were averaged, and the contact pair showing the largest average focal IED (i.e. the most active contact pair) was chosen as the contact pair of interest (COI) (see Table 1 for details on the anatomical location of each COI); this study is focused on the BOLD changes in the immediate vicinity of the most active contacts (see Voxels of interest section).

**Fig. 2:**
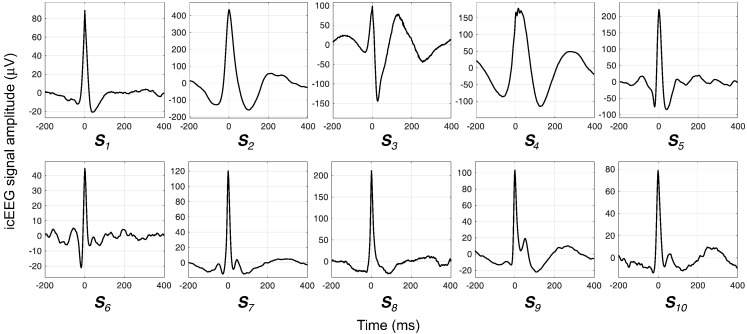
Average IED for each set of events of interest (***S***_*COI*_).

Since the BOLD changes in the immediate surroundings of a particular COI are expected to reflect all neuronal activity captured by the COI, all IED classes that shared the COI were grouped in a unique set of events of interest, called the ***S***_*COI*_. For example, let the contact pair # 1 be the COI; **A** the focal IED class observed at contact pairs # 1 and 2; and **B** the regional IED class observed at contact pairs # 1, 2, 12 and 13, such that **A** and **B** constitute all IED classes that involve contact pair # 1. In this case, events in **A** and **B** are taken to form the set of events of interest: *S* = {**A** ∪ **B**}. Through this process, IED were grouped in 10 ***S***_*COI*_, across all patients (see Table 1 for details on the IED classes modelled and numbers of IED that constitute each ***S***_*COI*_; see Fig. 2 for the average IED for each *S*_*COI*_).

The icEEG contacts coordinates used in the BOLD analysis were obtained in two steps: the creation of a patient-specific contacts mask; and the clustering of the voxels representing the contacts, together with the computation of the centres of mass (CM) of these clusters. The contacts mask was created by thresholding the intensity of an up-sampled version of the original CT, to isolate the voxels with the highest intensities (contact-voxels) from those with the lowest intensities (head tissues, CSF, and head-surrounding space). The threshold value was chosen by trial-and-error, so that all contacts were represented by a group of voxels large enough to be detected after the two co-registrations (using the SPM12 toolbox (www.fil.ion.ucl.ac.uk/spm/software/spm12/)) that followed: the contacts mask was, first, co-registered in the sMRI space, and, then, in the EPI space, and the nearest neighbour criterion was used to resample the images in both cases. The second step consisted in clustering the contact-voxels (in the EPI space) and finding the coordinates of the CM of these clusters using an in-house Matlab routine that did not require any prior information regarding the number of contacts or their positions. This routine consists of: computing the distances between each and every other contact-voxel; sorting these distances, for each contact-voxel, to find its nearest neighbours, which we defined to be the contact-voxels that are at most 4 mm apart from it; forming clusters composed of each contact-voxel and its nearest neighbours; finding the coordinates of the CM of every cluster and aggregating the CM with equal coordinates, therefore reducing the dimension of the problem - the resulting CM becomes the new contact-voxels; repeating the previous instructions, decreasing, each time, the diameter of the cluster in 0.25 mm, which leads to a unique set of coordinates {x,y,z} per contact. Once the coordinates of every contact were known, the contacts were plotted in a 3D representation, and visually labelled using the patient's implantation scheme and clinical notes as reference.

### IED parameterisation

**Fig. 3:**
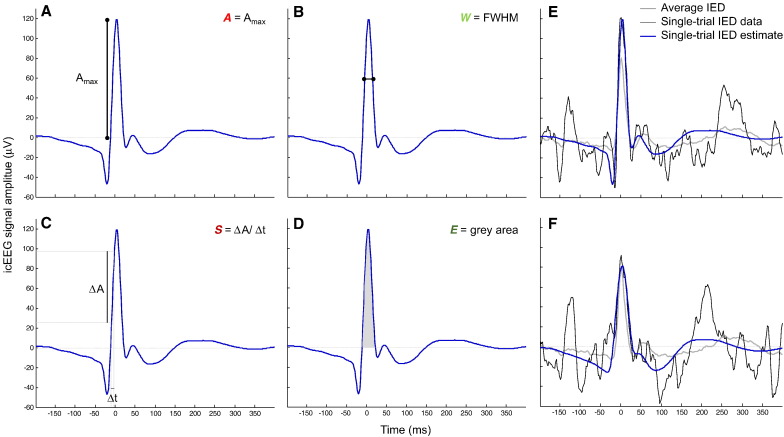
Sharp wave morphology-based features (A-D): A amplitude (A), width (B), slope of the rising phase (C), energy (D), overlaid on an example of a single-trial IED estimate. Single-trial IED estimates examples (E-F). The original IED is displayed in black, the average IED is displayed in grey, and the estimated IED is displayed in blue.

First, the EEG was high-pass filtered (low-cut-off at 3 Hz) and segmented into IED epochs of 600 ms duration, starting 200 ms before the IED marker. For each event, in any given ***S***_*COI*_, four sharp wave morphological features (see Fig. 3) and one sharp wave spatial field extent feature were estimated, as described below. These features were estimated from single-trial IED estimates rather than from the raw IED; the reason why is described below.

The IED sharp wave amplitude (*A*) was computed as the voltage value at the peak within a 40 ms-wide window, centred at the maximum of the averaged IED (Fig. 3 A). The sharp wave width (*W*) was computed as the full-width at half maximum:

(1) $$W=t_{{\left(V=0.5A\right)}^{+}}-t_{{\left(V=0.5A\right)}^{-}\;}$$

where *t*_(*V* = 0.5*A*)^−^_ and *t*_(*V* = 0.5*A*)^+^_ represent the points preceding and following, respectively, the sharp wave peak at which the signal is equal to 50% of *A* (Fig. 3 B). The slope of the rising phase of the sharp wave (*S*) was computed as the ratio:

(2) $$S=\frac{0.8A-0.2A}{t_{\left(V=0.8A\right)}-t_{\left(V=0.2A\right)}}$$

where *t*_(*V* = 0.2*A*)_ and *t*_(*V* = 0.8*A*)_ represent the points preceding the IED peak at which the signal is equal to 20% and 80% of *A*, respectively (Fig. 3 C). The sharp wave energy (*E*) was computed as the area under the curve of the estimated IED over the interval [*t*_(*V* = 0)^−^_, *t*_(*V* = 0)^+^_], where *t*_(*V* = 0)^−^_ and *t*_(*V* = 0)^+^_ represent the points preceding and following, respectively, the sharp wave peak at which the signal first crosses 0 (Fig. 3 D).

The IED spatial field extent (*SFE*) was estimated as the sum of the absolute value of the Pearson correlation coefficients between the epoch time courses from the COI and remaining contacts:

(3) $$\mathrm{SFE}=\sum_{c\epsilon C\backslash \left\{\mathrm{COI}\right\}}\left|\mathrm{corr}\left(e_{\mathrm{COI}},e_{c}\right)\right|$$

where *C* represents all contacts, *e*_*COI*_ is the epoch time course from the COI, *e*_*c*_ is the epoch time course from the contact *c*, and *corr*(,) stands for the Pearson correlation coefficient.

The morphological features were estimated using the EEG analysis toolbox STEP1 (http://iannettilab.webnode.com; Hu et al., 2011); the spatial field extent was estimated with a Matlab routine developed by us. In brief, the STEP1 processing consists of (1) computing the (COI-specific) average IED; (2) generating a variability matrix that models the variability of the latency and morphology across events (see below for details on how the variability matrix is built); (3) performing a principal component analysis (PCA) on this variability matrix; (4) using the three principal components (PC) that explain the most variance of the variability matrix as the basis set of a linear model, which is then used to obtain the single-trial IED estimate of a raw event. We chose to use a PCA-based method to obtain the single-trial IED estimates to overcome issues concerning the use of the raw signal; most importantly, the strong dependence of the accuracy of the feature estimate on the SNR of the raw signal. Let us, for instance, take the case shown in Fig. 3 F; the energy estimate using the raw signal (black curve) would be contaminated by the artefact peaking 25 ms before the sharp wave peak because the feature energy is computed as the area under the signal curve, over the interval [*t*_(*V* = 0)^−^_, *t*_(*V* = 0)^+^_], where *t*_(*V* = 0)^−^_ and *t*_(*V* = 0)^+^_ represent the points preceding and following the sharp wave peak, respectively, at which the signal first crosses 0. We chose to use the first three PC to compute the single-trial IED estimates given the way in which the variability matrix is built (by time shifting and changing the width of the (COI-specific) average IED (Hu et al., 2011)). In brief, the variability matrix is an array of multiple plausible synthetic IED, i.e., a basis set, derived from each (COI-specific) average IED and representing every combination of the following manipulations: shifting (by − 50 to + 50 ms in steps of 5 ms) and changing the width (by a compression factor ranging from 1 to 2, in steps of 0.05) of the (COI-specific) average IED, in relation to each single-trial IED. Therefore, the variability matrix is a set of base functions that differ in their shape, and can be linearly combined to create each single-trial IED estimate. The PCA on the variability matrix is performed to identify the three PC that explain most of the variance of the events shape variability. By linearly combining these PC, we obtain (COI-specific) IED estimates, that are fitted to each raw IED, and from which we can quantify the features of interest. Note that the amplitude variability was not explicitly modelled in the variability matrix but it is captured by the component's weights that result from the PCA. The three PC that explain most variance represent the average event, its temporal derivative, and its temporal dispersion (as shown in Hu et al. (2011)) because construction of the variability matrix is explicitly based on the events shape variability. Note that STEP1 was designed to remove the nefarious effects of noise, and therefore to reduce the proportion of variance which represents noise (i.e. overfitting) by choosing a basis set that captures well the key features of waveforms that most neurophysiologists would recognise as IED.

The mean and standard deviation of each IED-derived feature are presented in Table 1.

### Models of IED-related BOLD changes

For each ***S***_*COI*_, a total of six models of BOLD changes were estimated: M_O_, M_OA_, M_OW,_ M_OS_, M_OE_, and M_OSFE_, corresponding to the following effects of interest respectively: IED onsets alone, IED onsets and amplitude, IED onsets and width, IED onsets and slope, IED onsets and energy, and IED onsets and spatial field extent. Let D_O_, D_OA_, D_OW,_ D_OS_, D_OE_, and D_OSFE_, be the respective design matrices of these models: D_O_, the design matrix of the basic model, comprised IED onset times convolved with the canonical haemodynamic response function (HRF) (regressor O), and the following confounding effects (regressors C): 24 movement related confounds (6 realignment parameters, and their Volterra expansion (Friston et al., 1996)), and 6 fMRI physiological noise related confounds (Tierney et al., 2015). Each of the design matrices, corresponding to a feature, comprised the respective IED feature convolved with the canonical HRF as a modulatory effect of the amplitude of the stick functions placed at the IED onset times, such that D_OA_ = [A D_O_]; D_OW_ = [W D_O_]; D_OS_ = [S D_O_]; D_OE_ = [E D_O_]; and D_OSFE_ = [SFE D_O_]. For example, M_OW_ was defined as:

(4) $$y_{i}=\beta_{Oi}\times \left(O⊗\mathrm{HRF}\right)+\beta_{Wi}\times \left(W⊗\mathrm{HRF}\right)+\beta_{ci}C+\epsilon_{i}$$

where $y_{i}$ is the time course of the amplitude of the BOLD signal for the voxel *i*, × is the multiplication symbol, *O* is the sharp wave onset times regressor, ⊗ is the convolution symbol, *HRF* is the time course of the canonical HRF, *W* is the sharp wave width regressor, *C* is the confounding effects matrix, and *ϵ*_*i*_ is the error for the voxel *i*.

#### Variance explained quantification

To quantify the amount of BOLD signal (y) explained by a given model M, the coefficient of determination adjusted for the number of degrees of freedom, *R*^2^*adj*, was computed as:

(5) $$R^{2}\mathrm{adj}\left(M\right)=1-\frac{T-1}{T-P-1}\frac{\sum_{i=1}^{T}{\left(y_{i}-y_{i}^{\prime }\right)}^{2}}{\sum_{i=1}^{T}{\left(y_{i}-\overset{̅}{y}\right)}^{2}}$$

where *T* is the number of fMRI scans, *P* is the number of regressors in the model, $y_{i}$ and $y_{i}^{\prime }$ are respectively the $i^{\mathrm{th}}$ values of y and $y^{\prime }$ (the estimation of y obtained with M), and $\overset{̅}{y}$ is the temporal average of y. *R*^2^*adj* was chosen because it takes the number of degrees of freedom of the model into account, and expresses the degree to which the additional regressor is capable of explaining more variance than what would be expected by chance (if a random regressor was included).

To quantify the amount of BOLD signal, y, explained by a given regressor or set of regressors R, in addition to a regressor or set of regressors O, we computed the variance explained by R, VE(*R*), which corresponds to the difference between the *R*^2^*adj* obtained for the more complete model comprising both the set of regressors O and R, M_OR_, and the *R*^2^*adj* obtained for the simpler (and nested in M_OR_) model comprising only the regressor O, M_O_ (Bianciardi et al., 2009a, Shmueli et al., 2007), such that:

(6) $$\mathrm{VE}\left(R\right)=R^{2}\mathrm{adj}\left(M_{\mathrm{OR}}\right)-R^{2}\mathrm{adj}\left(M_{O}\right)$$

#### Voxels of interest

All models (M_OA_, M_OW,_ M_OS_, M_OE_, and M_OSFE_) were estimated using SPM12 (http://www.fil.ion.ucl.ac.uk/spm). Let us call the point halfway between the two contacts constituting the COI, the point of interest (POI). The voxels within a distance of 10 mm from the POI and that were significantly correlated (*p* < 0.05, uncorrected) with a positive or negative linear combination of the 2 regressors of interest (O + A, O + W, O + S, O + E, and O + SFE), found using the t-contrasts [1 1 0 …] (pBOLD voxels) or [−1 −1 0 …] (nBOLD voxels), respectively, were considered of interest. The variance explained was averaged across the pBOLD and nBOLD voxels, separately.

## Results

**Fig. 4:**
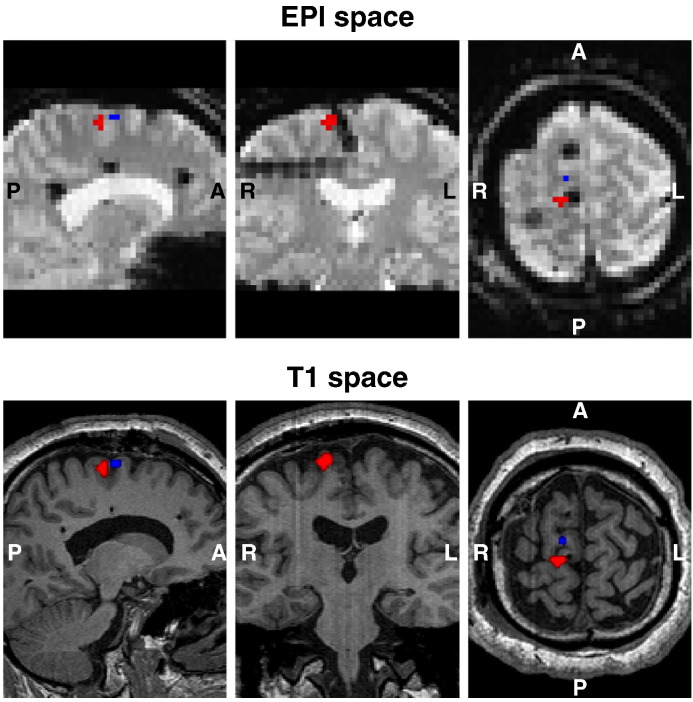
Representative example (patient ID: B, events of interest set: *S*_*2*_, see Table 1) of IED onset and width related BOLD changes (*p* < 0.05, uncorrected), within a 10 mm radius sphere centred at middle distance between the pair of icEEG contacts that shows the largest average IED (COI: PSMA 3–4). Positive BOLD changes (pBOLD) are showed in red, and negative BOLD changes (nBOLD) are showed in blue.

**Fig. 5:**
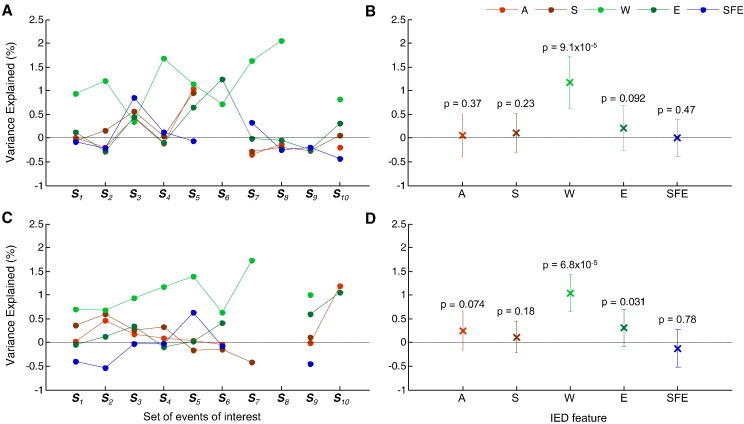
Variance explained (VE) results. Experiments level analysis: Average VE values, by modulatory feature; for the set of voxels comprised in pBOLD (A), and in nBOLD (C). Group level analysis: Average VE and respective standard deviation values, by modulatory feature; for the set of voxels comprised in pBOLD (B), and in nBOLD (D). Undefined values correspond to cases where no voxel had a large enough t-value to survive the threshold used (*p* < 0.05, uncorrected).

Representative examples of voxels of interest are presented in Fig. 4 (pBOLD in red; nBOLD in blue). Average *VE* values, for each IED feature, are shown in Fig. 5. The average *VE* values for IED feature *W* were above 0 in 9/10 IED sets for the BOLD increases; and 8/10 for the BOLD decreases (Fig. 5 A and C; Fig. S1 for event-by-event plots of BOLD signal amplitude vs sharp wave width (*W*)). This effect was statistically significant at the group level (Fig. 5 B and D). The feature *E* showed a trend towards explaining more variance (*p* = 0.09 for the BOLD increases; and *p* = 0.03 for the BOLD decreases) (Fig. 5 B and D).

## Discussion

We investigated the individual capability of four sharp wave morphological (amplitude, width, slope of the rising phase, and energy) and one spatial field extent features to explain variance of the amplitude of the co-localised BOLD signal that was not explained by the sharp wave onset times. Among these features, the width was the only one found to explain a significant amount of additional variance, suggesting that the amplitude of the BOLD signal depends more on the duration of the underlying field potential than on the degree of neuronal activity synchrony.

Previous work using a pharmacologic animal model of epilepsy found significant positive correlations between the amplitude and duration of epileptic LFP sharp waves and the amplitude of CBF changes, simultaneously recorded with laser-Doppler flowmetry (Geneslaw et al., 2011). A metabolic-hemodynamic model proposed by Sotero and Trujillo-Barreto, 2007, Sotero and Trujillo-Barreto, 2008, foresees amplitude, duration, and area under the excitatory curve of the sharp wave as good predictors of the amplitude of the BOLD signal (Voges et al., 2012). In the standard model of the BOLD effect, an increase in neuronal activity induces an increase in CBF, which provides more oxygen and glucose to the tissues; if the increase in CBF exceeds the simultaneous increase in oxygen consumption, the local concentration of deoxyhaemoglobin decreases and the intensity of the BOLD effect increases (Buxton, 2012). Due to the likely coupling between CBF and BOLD changes (Carmichael et al., 2008), the principal finding of this study -the duration of the sharp wave correlates significantly with the amplitude of the co-localised BOLD signal- is in line with both Geneslaw et al. (2011) and Voges et al. (2012).

### Sharp wave width neurophysiological correlates and the BOLD signal

Any type of transmembrane current across an excitable membrane contributes to an extracellular field potential that can be measured as LFP or icEEG. This field potential is the superposition of all ionic processes, ranging from fast action potentials to slow fluctuations in glia. All electrical currents in the brain superimpose at any given point in space to yield a (differential) potential, at that location. Therefore, any transmembrane current, irrespective of its origin, leads to an extracellular voltage deflection, whose characteristics depend on the proportional contribution of the multiple sources and properties of the brain tissue (Buzsaki et al., 2012). The LFP signal amplitude and spatiotemporal width are known to be markedly shaped by the impinging pattern of postsynaptic currents and membrane characteristics (Reimann et al., 2013). Therefore, the sharp waves width is likely to reflect the following aspects: (1) the duration and synchrony of excitatory PSP (EPSP), (2) the presence of inhibitory PSP (IPSP), and (3) the time constants of neurons. In particular, the sharp wave width is likely to reflect the distance between sources and field potential recording sensors (Gold et al., 2006); a larger distance can be associated with a lower degree of synchrony across multiple EPSP, which can sum and result in a wider (longer lasting) field potential, depending on the cells spatial arrangement. Hence, our main finding suggests that the BOLD signal amplitude is predicted by the duration of the underlying field potential, likely to reflect the sources geometric arrangement in relation to the recording sensors.

The sharp wave width effect was observed in voxels at which BOLD was either increased or decreased in relation to sharp wave onsets and widths; i.e. the sharp wave width explains additional variance of the BOLD signal amplitude in voxels where a linear combination of the events onsets and widths is positively or negatively correlated with the BOLD signal amplitude. Both IED onsets - related BOLD signal increases and decreases have been reported (Benar et al., 2006, Gotman et al., 2006, Grouiller et al., 2010, Jacobs et al., 2014, Lemieux et al., 2008, Moeller et al., 2009, Pittau et al., 2013, Salek-Haddadi et al., 2006). However, the mechanisms underlying BOLD signal decreases are not completely understood: they may result from (1) neuronal activity decreases (Shmuel et al., 2006) and associated CBF decreases (Carmichael et al., 2008), or (2) neuronal activity increases that lead to tissue oxygen consumption increases that exceed the simultaneous CBF increases, resulting in local deoxyhaemoglobin concentration increases (Schridde et al., 2008). Finding that a significant amount of the variance of BOLD signal decreases was explained by the sharp wave width suggests that BOLD decreases may not be necessarily associated with neuronal activity decreases (hypothesis (1)); our main finding and those of Geneslaw et al. (2011) favour hypothesis (2).

### Sharp wave amplitude neurophysiological correlates and the BOLD signal

The LFP, icEEG, and scalp EEG signals represent extracellular field potentials primarily originated by postsynaptic activity, integrated over different volumes (Creutzfeldt et al., 1966a, Creutzfeldt et al., 1966b, Klee et al., 1965, Niedermeyer and Lopes da Silva, 1999). The amplitude of these signals depends on the geometric arrangement of the active cells, within each element volume, as well as on the degree of synchrony among the multiple element volumes, over larger distances (Einevoll et al., 2013). Due to the different nature of the EEG and BOLD signals, decoupling between them is to be expected to some degree (Nunez and Silberstein, 2000). For instance, active pyramidal cells are expected to be associated with a high metabolic demand, due to their action potentials firing frequency (Connors and Gutnick, 1990), and with large current dipoles, which result from the sum of many “open-field generators”. However, the amplitude of the BOLD signal may be independent of the geometric arrangement of active cells and equally sensitive to synchronous and asynchronous activity (Nunez and Silberstein, 2000). For instance, BOLD changes may be coupled to neuronal signalling processes rather than to energy demand (Attwell and Iadecola, 2002) or neuronal activity synchronisation. Hence, the sharp wave amplitude may or may not be a good predictor of the BOLD signal amplitude.

While Benar et al. (2002) only found a low (not significant), positive correlation between the square root of the EEG and BOLD signals in the time range of the interictal event (sharp and slow waves taken together), LeVan et al. (2010) found significant correlations between the amplitudes of scalp EEG sharp waves and BOLD changes in the SOZ, but not in distant regions. One of the reasons why we did not find a significant correlation between the amplitudes of icEEG sharp waves and co-localised BOLD changes may be related with the spatial scale of our electrophysiological measurements. For instance, Keller et al. (2010) found that sharp waves recorded with microelectrodes in humans could occur with relatively sparse neuronal participation. Furthermore, sharp waves on icEEG can be generated by much smaller neuronal populations than sharp waves on scalp EEG (Cooper et al., 1965, Ebersole, 1997, Nunez and Silberstein, 2000); in particular, the first may be generated by a synchronous, albeit small, neuronal population, whose activity does not involve a large increase in metabolic demand.

### IED spatial field extent neurophysiological correlates and the BOLD signal

IED generation is thought to reflect a dynamic and complex network phenomenon, which is not yet completely understood (Keller et al., 2010). However, assuming that IED simultaneously observed at multiple icEEG contacts are comparable to LFP correlated across large distances (> 0.2 mm), and noting that the LFP field extent is mainly dependent on the spatial extent of the surrounding, synchronised sources of neuronal activity (Einevoll et al., 2013), we may hypothesise that IED are generated by either multiple, synchronous neuronal populations, or a single population, whose activity (instantaneously) spreads to multiple contacts, through volume conduction. Since the BOLD signal amplitude does not seem to strongly reflect the sharp wave spatial field extent, it may also not reflect the volume of the surrounding, synchronised sources.

### Methodological aspects and technical limitations of this work

This study is based on the comparison of two models, one nested in the other. More specifically, we estimate the amount of variance explained by each feature, quantified event-by-event, in addition to the event onset; the latter being the standard way to model IED. Irrespective of the absolute amount of variance explained by any of these features, this approach allowed us to rank them according to the amount of variance explained in addition to a common reference.

We chose to use the simplest possible model for the relationship between the amplitude of the BOLD signal and each sharp wave feature, implying that the former is linearly proportional to the latter, through convolution with a fixed haemodynamic kernel, in this case, the canonical HRF. This choice was based on three main reasons. Firstly, we had to explicitly and specifically test for BOLD changes related with the EEG-derived features because the primary purpose of this study was to better understand the neurophysiological correlates of the BOLD signal. The canonical HRF simplicity allowed for a limited number of degrees of freedom and, therefore, a more straightforward and unambiguous interpretation of the results. In fact, the number of comparisons would increase dramatically if we had chosen to use a more flexible/complex HRF model, which we think is unwise given the relatively limited amount of data at hand. Moreover, the possibility of using other hemodynamic kernel does not invalidate our main finding: among all factors considered, only sharp wave width explained a significant amount of additional variance of the amplitude of the BOLD signal. Secondly, we wanted to be consistent with the previous fundamental studies on the local electrophysiological correlates of the BOLD signal (Goense and Logothetis, 2008, Magri et al., 2012, Nir et al., 2007, Scheeringa et al., 2011). Thirdly, although some studies of epileptic activity have raised the issue of the choice of the hemodynamic kernel (deviations from the canonical HRF shape have been observed, mostly in relation to generalised discharges or focal discharges in generalised syndromes (Beers et al., 2015, Grouiller et al., 2010, Masterton et al., 2010, Moeller et al., 2008)), others have found this variability to be less significant, particularly in relation to focal discharges, with deviants often remote from the presumed primary generator of epileptic activity (Lemieux et al., 2008, Proulx et al., 2014). This study was focused on BOLD changes in the immediate vicinity of the most active icEEG contacts, i.e., BOLD changes within a small volume of brain tissue not expected to exhibit different haemodynamic responses. Furthermore, reports of HRF shape variability are not limited to studies of epileptic activity; it has also been observed in relation to location in the healthy brain, using a relatively constrained basis set (Aguirre et al., 1998), and in relation to various normal stimuli (Grouiller et al., 2010, Handwerker et al., 2004). Analyses of exceptionally high SNR fMRI data of normal brain activation, using a totally unconstrained hemodynamic kernel basis set, have revealed a wide range of HRF shapes covering almost the entire brain, but with unknown biological meaning (neuronal vs vascular effects) for the deviant ones (Gonzalez-Castillo et al., 2012, Gonzalez-Castillo et al., 2014).

We, and others, have found that BOLD changes related to epileptic activity, including the statistical maximum, can be located remotely from the most active electrodes (Chaudhary et al., 2012, Flanagan et al., 2014, Kobayashi et al., 2009, Krakow et al., 1999). By focusing on the local relationship between IED features and BOLD signal amplitude, we ensure that the knowledge gained is more directly related to the generators rather than any potential propagation effects.

The apparent absence of BOLD changes despite the presence of neuronal activity, due to the limited spatial resolution of fMRI acquisitions, together with a diminished signal to noise ratio (SNR) in the surroundings of the metallic icEEG contacts, caused by magnetic susceptibility gradient-induced signal drop-out are limitations of this study. The quality of our fMRI data was quantified and discussed in the previous study, Carmichael et al. (2012). Notwithstanding that the % of signal loss varied across contacts depending on the electrode orientation relative to the MRI scanner axes (there were greater losses for contacts with a vector normal to the grid surface parallel to B_0_), the amplitude of the fMRI signal was generally around 70% of its whole brain average value at ~ 5 mm away from the icEEG contact and 100% at ~ 10 mm away from it (Carmichael et al., 2012). Our approach has been to use clinically certified electrodes, with sub-optimal imaging properties, and to scan at 1.5 T, which reduces health risks and the amount of signal drop-out compared to 3 T (Carmichael et al., 2012); we note that the development of electrodes with improved imaging properties and excellent electrophysiological recording characteristics, suitable for clinical use, represents an interesting challenge.

The lack of a strong correlation between the sharp wave amplitude and BOLD signal amplitude may be a consequence of these limitations because large sharp waves may be the reflection of highly local, synchronised activity (Einevoll et al., 2013) that is so spatially restricted that it cannot be captured by fMRI. Even so, we found a significant correlation between the sharp wave width and the BOLD signal amplitude because wider sharp waves may be the reflection of widespread, not perfectively synchronised EPSP, that sum and give rise to a wider field potential. A larger number of sets of events of interest (*S*_COI_) could reveal that other sharp wave features (for example, amplitude) are also significantly correlated with the amplitude of the BOLD signal. Nevertheless, the significant effect of the sharp wave width would almost surely remain. We believe that our 10 *S*_COI_ are representative of interictal epileptic events in general; they were recorded from multiple brain regions (amygdale, anterior and posterior supplementary sensorimotor areas, posterior cingulate, superior, middle, and inferior frontal gyrus, and the lateral orbitofrontal area), and show relatively heterogeneous shapes (Fig. 2).

### Relevance to EEG-fMRI studies

Most EEG-fMRI studies of epileptic activity use IED onsets as the only EEG-feature of interest. As a supplementary analysis, we computed the variance explained by IED onsets in addition to confounds (i.e. we compared the residuals of the models [IED onsets + confounds] and [confounds]); we found average VE of 1.17% (pBOLD) and 1.21% (nBOLD). Therefore, the amount of BOLD signal variance explained by IED onsets, but not by confounds, is of the same magnitude as the variance explained by sharp wave widths, but not by IED onsets and confounds. Our VE values are comparable to studies of physiological noise, Jorge et al. (2013) and Bianciardi et al. (2009b), reporting average VE values (within grey matter masks) of the order of 1% and 2%. Therefore, modelling sharp wave width variability, in addition to their onsets, is likely to improve the BOLD sensitivity related to epileptic activity; this may be relevant for scalp EEG-fMRI studies aiming to map the focus and/or propagation networks underlying epileptic activity.

### Relevance to neuroscience

Sharp waves have been observed in both pathological and healthy contexts (Sullivan et al., 2011). In particular, physiological sharp waves were recorded in the hippocampal CA1 stratum radiatum of healthy rodents having minimal interaction with their environment (during immobility, consummatory behaviours, or slow-wave sleep) (Buzsáki et al., 1983, Suzuki and Smith, 1987), and of healthy macaques at an inactive/drowsy-or-sleeping behavioural state (Skaggs et al., 2007). This suggests that our main finding may be relevant for non-epileptic tissues; the BOLD signal amplitude may be generally dependent on the duration of the underlying field potential.

## Conclusion

We compared a number of epileptic sharp wave features (amplitude, width, energy, rising slope, and field extent) in terms of their individual capability to explain variance of the co-localised BOLD signal that was not explained by sharp wave onset times alone; we found that the width was the only one explaining a significant amount of additional variance. This suggests that the amplitude of the BOLD signal depends more on the duration of the underlying field potential than on the degree of neuronal activity synchrony.

The following is the supplementary data related to this article.

Supplementary Fig. S1 Event-by-event relationship between BOLD signal amplitude and sharp wave width (*W*), for each set of events of interest (*S*_*COI*_). The slopes of the red and blue lines are equal to *βw* (see Eq. (4)), for the pBOLD and nBOLD voxel sets, respectively. The corresponding Pearson correlation coefficients are shown on the top right of each plot; * indicates significant correlations (*p* < 0.05). Supplementary Fig. S1

## Figures and Tables

**Table 1:** Electrophysiological data and effects of interest for BOLD modelling: types of icEEG electrodes used and number of IED events included in the BOLD models. D: depth electrode contacts, E: electrocorticography (ECoG) contacts, R: right, L: left, A: anterior, M: medial, P: posterior, I: inferior, and S: superior.

Patient ID	A	B			C		D		E	F
Type of epilepsy	Temporal lobe epilepsy	Frontal lobe epilepsy								
Summary of the icEEG implantation	- R and L amygdalae (R A) - R and L hippocampi	- R A and P insula - R A (R ASMA) and P (R PSMA) supplementary sensorimotor areas - R A, M and P cingulum (P C)			- L superior (SFG), middle (MFG) and inferior frontal gyrus - L precentral gyrus - L central sulcus and part of postcentral sulcus - L superior frontal sulcus - L postcentral regions		- L frontal lobe (laterally and inferiorly) - L M (MFG) and I (IFG) frontal gyrus - L frontal pole		- L frontal lobe (laterally and inferiorly) - L M (MFG) and I (IFG) frontal gyrus - L temporal lobe	- L frontal and parietal convexity - L frontal pole - L S frontal gyrus (SFG) - L I frontal gyrus - L mesial frontal surface
Number of icEEG contacts	Five 6-contact depths	Two 6-contact depths, three 8-contact depths, two 10-contact depths			One 8 × 8 contact grid, two 4-contact depths, one 2 × 8 contact grid		One 8 × 8 contact grid, one 2 × 8 contact grid, two 6-contact depths, two 6-contact strips		One 6 × 8 contact grid, two 2 × 8 contact grids, one 4 × 8 high-density contact grid, two 6-contact strips, two 6-contact depths	One 8 × 8 contact grid, one 2 × 8 contact grid, one 8-contact strip, one 6-contact strip, one high-density 4 × 8 contact grid
Number of fMRI sessions	2	2			1		1		1	2
Type of contact	D	D			E		D	E	D	E
Distance between the contacts (mm)	5	10	5	10	10	10	10	10	10	10
Number of focal IED classes	1	3			2		2		1	1
Location of COI	R A	R P SMA	R A S MA	R P C	L P SFG and MFG	L P SFG and MFG	L IFG and MFG	L lateral orbitofrontal	L IFG and MFG	L SFG
Irritative Zone	R and L temporal lobes	R ASMA and R PSMA		R I parietal and M frontal gyrus	L P SFG and MFG		L IFG and MFG	L lateral orbitofrontal	L IFG and MFG	L SFG (lateral and medial)
IED classes	Focal plus more widespread	Focal	Focal plus more widespread					Focal	Focal plus more widespread	Focal
Set of events of interest	*S*_1_	*S*_2_	*S*_3_	*S*_4_	*S*_5_	*S*_6_	*S*_7_	*S*_8_	*S*_9_	*S*_10_
Number of IEDs at the COI	1630	209	470	253	397	613	993	194	887	191
IcEEG feature (mean ± standard deviation)										
Amplitude (μV)	71.15 ± 91.14	368.60 ± 395.52	78.62 ± 62.34	139.43 ± 241.95	101.38 ± 72.52	19.40 ± 28.51	47.11 ± 40.67	78.73 ± 66.86	41.18 ± 26.64	66.19 ± 53.36
Slope (μV / s)	4.67 ± 6.96	12.68 ± 18.04	6.45 ± 5.38	4.94 ± 8.75	7.85 ± 6.09	2.48 ± 3.77	3.77 ± 3.22	6.15 ± 5.68	3.46 ± 2.25	5.83 ± 4.96
Width (FWHM) (ms)	23.19 ± 6.83	40.89 ± 10.95	19.47 ± 4.08	48.68 ± 21.24	10.46 ± 2.10	8.91 ± 4.03	9.88 ± 2.90	10.81 ± 2.57	11.29 ± 4.49	21.30 ± 6.33
Energy (μV s)	1.78 ± 2.27	16.00 ± 16.51	1.48 ± 1.33	7.78 ± 12.97	2.06 ± 1.48	0.31 ± 0.49	1.01 ± 1.01	1.71 ± 1.58	1.03 ± 0.66	1.40 ± 1.27
Spatial field spread (a.u.)	7.06 ± 1.61	14.85 ± 2.88	13.24 ± 2.47	14.18 ± 2.18	10.93 ± 2.76	9.45 ± 2.42	10.27 ± 1.70	9.63 ± 2.00	7.38 ± 1.61	17.32 ± 3.59
