# Supplementary figures and images for: A study of the electro-haemodynamic coupling using simultaneously acquired intracranial EEG and fMRI data in humans
Source: Neuroimage. 2016 Nov 15;142:371–80. doi: 10.1016/j.neuroimage.2016.08.001 (PMC5102699; doi:10.1016/j.neuroimage.2016.08.001)

BOLD signal amplitude (a.u.)

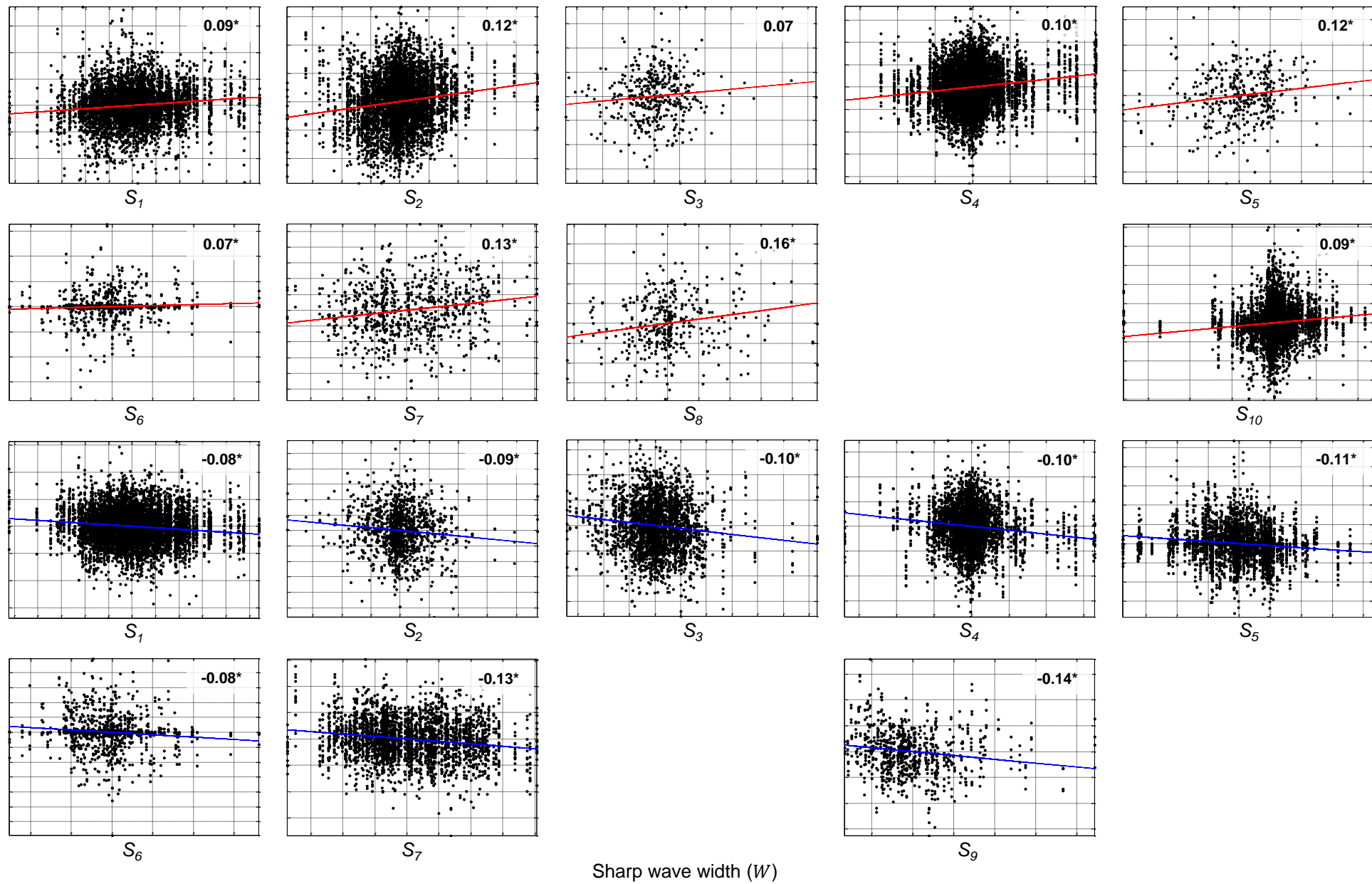

Supplement: Supplementary Fig. S1 — Event-by-event relationship between BOLD signal amplitude and sharp wave width (W), for each set of events of interest (SCOI). The slopes of the red and blue lines are equal to βw (see Eq. (4)), for the pBOLD and nBOLD voxel sets, respectively. The corresponding Pearson correlation coefficients are shown on the top right of each plot; * indicates significant correlations (p < 0.05). [file mmc1.pdf]
